# Passengers as Pathways: Behavioral Evidence on Travelers’ Knowledge of African Swine Fever Introduction Through Pork Products

**DOI:** 10.3390/vetsci13020194

**Published:** 2026-02-17

**Authors:** Daniela Mandas, Giulia Murgia, Katia Usai, Riccardo Bazzardi, Gaia Muroni, Stefano Cappai, Annamaria Coccollone, Federica Loi

**Affiliations:** 1 Istituto Zooprofilattico Della Sardegna, Sede Diagnostica Territoriale di Cagliari, Strada St. 130, 09030 Elmas, Italy; daniela.mandas@izs-sardegna.it (D.M.); giulia.murgia@izs-sardegna.it (G.M.); annamaria.coccollone@izs-sardegna.it (A.C.); 2 Istituto Zooprofilattico Della Sardegna, Osservatorio Epidemiologico Veterinario Regionale, Via XX Settembre 13, 09125 Cagliari, Italy; katia.usai@izs-sardegna.it (K.U.); gaia.muroni@izs-sardegna.it (G.M.); stefano.cappai@izs-sardegna.it (S.C.); 3 Istituto Zooprofilattico Della Sardegna, Via Vienna 9, 07100 Sassari, Italy; riccardo.bazzardi@izs-sardegna.it

**Keywords:** African swine fever, social science, early detection

## Abstract

Increased international trade and tourist flows are key factors in the introduction of transboundary animal diseases such as African swine fever (ASF). Despite the availability of sufficiently detailed data on legal commercial trade, the movement of pork products intended for personal consumption is insufficiently reported and difficult to track when carried in passenger luggage, and may act as an introduction pathway. In this study, we analyze the risk of ASF reintroduction in Sardinia, an island that has recently achieved disease-free status after a long epidemic. An anonymous questionnaire on travelers’ awareness of ASF and their food transportation habits was administered at the main Sardinian airports between July and December 2025, and a total of 6525 responses were received. The results show a low level of knowledge regarding ASF, with approximately 95% of respondents stating they were unaware of the disease, while almost 10% of travelers reported having transported meat or other pork products, often originating from countries where the virus is circulating. Our investigation highlights how passenger flows, especially during the seasonal peak, could act as a vector for potential disease reintroduction through the transport of pork products. The results stress that the adoption of behavioral survey tools is essential to supplement traditional surveillance systems, highlighting the need to improve targeted communication strategies at both ports and airports in order to reduce the risk of virus reintroduction and protect regional livestock.

## Simple Summary

Though it does not pose a risk to human health, African swine fever is deadly to pigs and wild boar and can lead to severe economic losses; tourists can unintentionally contribute to its spread by transporting pork products during international travel. Countries with high tourism rates may therefore be exposed to new risks of introduction. In this study, an anonymous questionnaire was administered at airports in Sardinia (Italy) to explore travelers’ awareness of the disease and their food transport-related habits. The results showed that knowledge of African swine fever among international travelers was very limited. Approximately one in ten travelers reported transporting pork products for personal consumption, often without being aware of the possible consequences. Tourist-related travel may further increase the risk of African swine fever when staying in rural or natural areas where wild boar are present; the improper disposal of food waste may lead to contact between contaminated products and wildlife. These findings highlight the importance of improving communication and awareness at airports to help prevent the reintroduction of animal diseases in order to protect livestock, wildlife, and food systems.

## 1. Introduction

Several factors have led to the introduction or emergence of new animal diseases and zoonoses: increased international trade in live animals and food products, the movement and mixing of wild animal species, and, in the case of vector-borne diseases, the effects of climate change. As such, demand is growing for rapid and effective surveillance and response systems that ensure the optimal use of limited human and financial resources [1,2]. The recent introduction of diseases such as African swine fever (ASF) and lumpy skin disease into Italy has highlighted the country’s exposure to infections originating from outside its borders; both its geographical location in the center of the Mediterranean and the trade flows of live animals and animal products are contributing factors [3,4].

Using trade flow data, risk assessment models, and scenario-based approaches, several studies have qualitatively and quantitatively estimated the risk of ASF introduction into different countries through the legal trade of pork and its related products [5,6,7]. Epidemiological analyses have consistently identified that the movements of both live pigs and pig-derived products are major pathways in the long-distance spread of ASF virus (ASFV) [8,9,10]. While detailed data on live animal movements are generally available in national and international databases, information on the illegal trading and transportation of pork products for personal consumption (e.g., in passenger luggage on air or sea routes) remains largely unavailable, mainly due to a lack of standardized and accredited sources.

In parallel, a growing body of literature in the fields of public health, travel medicine, and infectious disease epidemiology has demonstrated the usefulness of administering questionnaires to travelers—particularly in airports and ports of entry—to assess their risk perception, knowledge of infectious diseases, compliance with preventive measures, and behaviors that may facilitate disease introduction [11]. Such studies have explored a wide range of human infectious diseases—including influenza, SARS, Ebola virus disease, and COVID-19—and antimicrobial-resistant infections, demonstrating that traveler awareness, origin, travel routes, and declared behaviors can be effectively captured through structured surveys conducted at points of entry or via digital tools [12].

In airports, questionnaire-based approaches have been used to evaluate travelers’ awareness of disease transmission routes, their adherence to biosecurity or hygiene recommendations, and their likelihood of engaging in behaviors associated with increased risk of pathogen spread [13]. These tools have also proven valuable for rapidly collecting large amounts of data from heterogeneous populations, especially since traditional surveillance systems are unable to capture individual-level behaviors related to travel and personal consumption practices [14].

However, despite the extensive use of questionnaires to investigate disease awareness and preventive behaviors among travelers, they have largely only been applied to human public health contexts [15]. To the best of our knowledge, no previous studies have specifically investigated links between air or sea travel, the transport of pork or pork-derived products for personal consumption, and the risk of (re)introduction of transboundary animal diseases such as ASF. This represents a critical gap in our current understanding of disease introduction pathways, particularly for regions characterized by high tourist flows and strong livestock–territory interactions.

In this context, Sardinia is a particularly relevant case study. The island has experienced a unique and long-lasting ASF epidemic, only achieving official disease-free status recently. Given its insular nature, its reliance on air and sea connections, and the large volume of incoming passengers relative to the resident population, Sardinia faces the risk of potential animal pathogen introduction through unregulated passenger-borne food products [16]. The aims of the present study are twofold: first, to raise awareness of the risk of ASF reintroduction in Sardinia, and second, to address existing information gaps by administering a questionnaire to air travelers that assesses their knowledge of ASF, travel patterns, and transportation of pork products for personal consumption. As such, this study provides novel, behavior-based evidence to support risk assessments and targeted prevention strategies at points of entry.

## 2. Materials and Methods

### 2.1. Study Area and Epidemiological Context of ASFV

Sardinia is an autonomous island region of Italy with a total area of 24,100 km^2^, divided into four provinces: Cagliari (a metropolitan area), Oristano, Sassari, and Nuoro. It is connected to the rest of Italy and Europe by three main airports (Alghero, Cagliari, and Olbia) and four main ports (Cagliari, Olbia, Arbatax, and Porto Torres). Sardinia is characterized by a low population density, equal to 69 hab./km^2^ (one and a half million inhabitants in total): the third-lowest of the Italian regions. Given its unspoiled landscape and many natural attractions, Sardinia is a popular tourist destination, especially in the summer. The regional data (https://www.sardegnamobilita.it/bollettino-arrivi-e-partenze) (access date: 2 February 2026) shows that the total number of tourists who arrived by air in 2025 was about five times the number of inhabitants (5,412,606), with about two and a half million arriving by sea. Most of the land in Sardinia is used for pasture and agriculture, of which 60.1% is used for farming, 34.1% for agriculture, and the remainder for wood cultivation. Sardinia specializes in sheep–goat breeding and, to a lesser extent, cattle and pig rearing (7° Agri-Census ISTAT, 2020. https://www.istat.it/statistiche-per-temi/censimenti/agricoltura/7-censimento-generale/risultati/; access date: 2 February 2026); swine husbandry has always been a secondary activity compared to the island’s other livestock production systems [17]. Sardinia has been affected by ASFV since 1978 and was finally declared to be disease-free in 2024. Its unique epidemiological context, which has previously been described elsewhere, made this a complex battle [17]. While the rest of Europe is affected by ASFV genotype II, the island of Sardinia was the only part of the continent affected by genotype I [18]. Most Sardinian domestic pig farms are focused on personal consumption and have familiar or working relationships with other farms, with only 5% being commercial farms [9]. The virus was detected in Sardinian domestic pigs for the last time in September 2018, while the last PCR-positive wild boar was identified in April 2019 [18]. In 2021, Sardinia’s status in relation to restrictions on ASF control measures changed, passing from the level of risk for countries included in Part IV (highest risk) to Part III of ASF risk areas [19], and in 2024, the Island was declared an area free from ASFV [20]. A long-distance introduction of ASF occurred in 2023; genotype II was detected in Nuoro province, likely linked to improper disposal of infected meat or meat products originating from mainland Italy [21]. The outbreak was brought under control immediately and no spread occurred. As Sardinia was endemic for ASF for more than 40 years, the importation and exportation of live swine or pork products were banned until disease-free status was achieved. Despite this, the island continues to record low exports/imports every year: in 2025, a total of 7340 live swine were imported, and a total of 11,153 live swine were exported to mainland Italy [22].

### 2.2. Questionnaire on Illegal Trade and Transportation of Pork Products for Consumption

In this study, we aimed to address the information gap with regard to the risk of air and sea passengers reintroducing ASF through the transportation of untracked pork products (tourist routes).

In the fields of social science and public health, visual communication tools such as posters and illustrated materials have long been used to both raise awareness and act as integral components of research design and data collection. Interactive and static posters and leaflets have been successfully employed to engage heterogeneous populations, stimulate participation, and convey risk-related information in a standardized and accessible manner [23]; to demonstrate how visual elements influence public understanding of health risks and preventive behaviors [24]; and to provide measurable effects on knowledge and awareness, supporting their role as legitimate methodological tools within health communication research [25,26].

Within this framework, the use of illustrative materials and visual supports at points of entry, such as airports, is consistent with established approaches in the social and behavioral sciences for improving public engagement and risk awareness while facilitating data collection in real-world settings.

**Figure 1:**
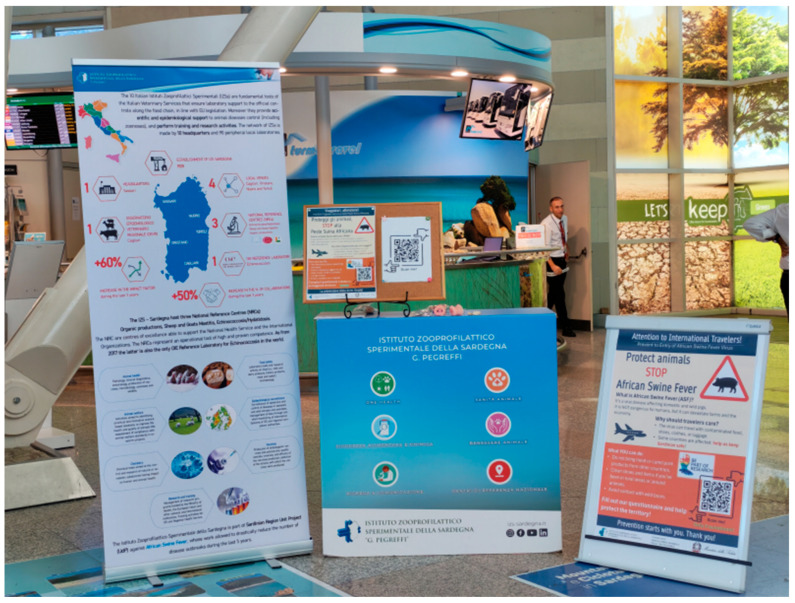
Logistics stand for administering questionnaires at Olbia Airport.

The experiment was conducted between 1 July and 31 December 2025. A six-item, single-choice questionnaire was administered on a voluntary and anonymous basis to passengers arriving at the main airports of Sardinia to assess their knowledge of ASF, their destination and origin, and whether they had ever transported processed pork products. Two methods of administration were used. In the first, questionnaires were delivered by operators present on site, particularly in the arrival areas. During administration, a stand was set up to attract the attention of passengers and provide further information (Figure 1).

**Figure 2:**
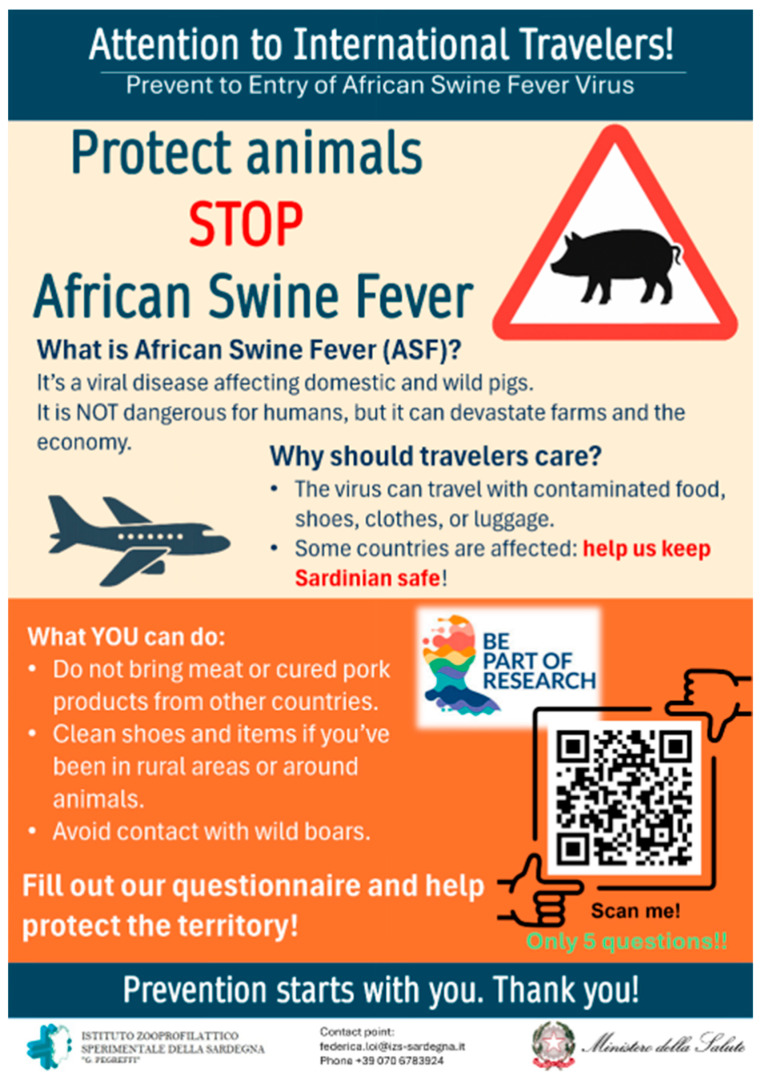
English version of the poster displayed at Alghero, Cagliari, and Olbia airports, including the QR code (https://docs.google.com/forms/d/1ymIvWQ5JSRKb0ET-Gb_oBgh8hy_WysVwvxFTthJFAwk/edit, access date: 2 February 2026) for completing the anonymous questionnaire.

Given the large number of passengers to be reached, it was decided to test a second administration method using QR codes. Posters were created to raise awareness of ASF, explaining the main risks associated with transporting meat or meat products and inviting participants to take part in the study by completing the questionnaire accessible via the QR code (Figure 2). Data from questionnaires completed using this method were stored on the online Google Forms (Google LLC, Mountain View, CA, USA), available online from 1 July to 31 December 2025. Both English and Italian versions of the questionnaire and posters were produced.

### 2.3. Study Design and Participants

This is a cross-sectional study conducted using an airport-based and web-based open survey. The study population included travelers who arrived in Sardinia by airplane during the summer–autumn season (July–December) in 2025. All participants, who had been recruited using “snow-ball” sampling, were required to provide their informed consent and agree to the collection and storage of their data for analysis and publication before taking part in the web survey. The survey was completely anonymous. Participants were made aware of the voluntary nature of their participation and the confidentiality of their information was assured.

### 2.4. Statistical Analysis

Answers provided during the study period (1 July to 31 December 2025) were downloaded from the Google Forms platform. The completeness and consistency of the collected data, which were stored in an ad hoc database, were evaluated. Given the qualitative nature of the data collected, all variables were summarized as frequencies and percentage. The final sample size was equal to the number of travelers who completed the questionnaire either online or with the expert operators on site. Furthermore, to validate the sample and determine if it reflected the general population of travelers who arrived in Sardinia during the study period, trend analysis was performed by comparing our data with that published by the Sardinian Airport Authorities. The total number of passengers arriving, their origin, the number of arrivals per season, and weekly trends were compared with the official overall data, available at https://www.sardegnamobilita.it/bollettino-grafici-portoaeroporto (access date: 2 February 2026).

Comparisons of qualitative variables were performed based on either the Chi-square test or Fisher’s exact test. In order to compare differences between quantitative variables, the Kruskal–Wallis nonparametric test was applied. A *p*-value < 0.05 was considered to be significant for all analyses. All statistical analyses were performed using R software (Version 4.5.2, R-Foundation for Statistical Computing, Vienna, Austria).

## 3. Results

**Figure 3:**
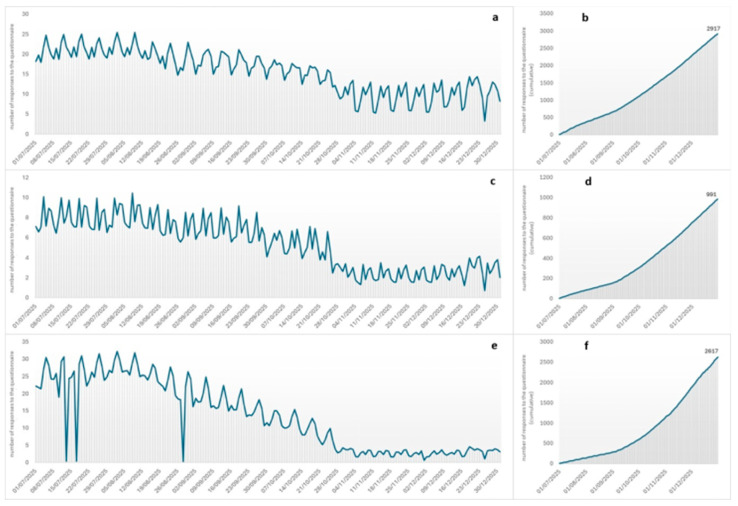
Number of questionnaire responses collected daily and cumulatively from 1 July to 31 December 2025 in (**a**,**b**) Cagliari, (**c**,**d**) Alghero, and (**e**,**f**) Olbia airports.

From 1 July to 31 December 2025, a total of 6525 anonymized answers were collected on the Google Forms page. The data mainly follows the typical non-normal distribution trend described by the Sardinian Airport Authorities (https://www.sardegnamobilita.it/bollettino-grafici-portoaeroporto; access date: 2 February 2026): during weekends (Friday, Saturday, and Sunday), a median of 40 responses/day were received [I-III quartile = 25–57], while during weekdays, the median was about 31 responses per day [I-III quartile = 16–47], showing a statistically significant difference (Mann–Whitney test: z-value = −3.711, *p*-value = 0.0002) (Figure 3). Statistically significant differences (Mann–Whitney test: z-value = 11.256, *p*-value < 0.0001) were also observed between seasons: most responses were recorded during the summer period (July–September) (4542, 70%) with an associated median of about 51 responses/day [I-III quartile = 43–55], as shown in Figure 3a,c,e.

Most of the data were collected in Cagliari Airport, where a total of 2917 (44.7%) travelers completed the questionnaire using the QR code, compared to 991 (15.2%) in Alghero Airport and 38.9% in Olbia Airport (Figure 3b,d,f). Only 80 (1.2%) travelers were interviewed by operators present at the arrival area in Olbia Airport.

The results of the 6525 anonymized responses to the six questions (Q1–Q6) were extracted from the Google Forms page and are reported question-by-question in Table 1. Data are presented in descending numerical order for ease of interpretation.

As expected, most travelers (5530) came to Sardinia on vacation (84.7%), while 914 (14%) came for work, and only 80 came to study or for other reasons (1.3%). In total, 62.8% (4094) of travelers who answered the anonymous questionnaire came from Western Europe, compared to 35.6% (2324) from Eastern Europe and only 1.6% (106) from Asia. No travelers from Africa, America, or Australia answered the questionnaire. Given Sardinia’s status as an island, it was not surprising that most respondents made a fly stop (3956, 60.6%).

Most responses were received from people who made a fly stop in or came from Italy (2802, 43%), Germany (1512, 23.2%), Belgium (616, 9.4%), Spain (590, 9.0%), the Czech Republic (466, 7.1%), France (338, 5.2%), Poland (178, 2.7%), Russia (18, 0.3%), and Switzerland (4, 0.1%).

Approximately 95% (6188) of the sample stated that they were unaware of ASF (Q5), while 9.7% (630) stated that they were transporting meat, sausages, or other pork products from their home country or from the country where their flight stopped (Q6).

By performing an in-depth analysis of the 5% (336) of respondents who stated they were aware of ASF, we found that 9.5% (56) arrived from Spain, 8.5% (128) from Germany, 7.7% from France, 4.5% (8) from Poland, 3.9% (110) from mainland Italy, and only 1.7% (8) from the Czech Republic. Furthermore, of this subgroup, 310 came to Sardinia on vacation, while only 26 came for work (5.6%) or to study (2.8%).

A further in-depth analysis was conducted on responses to Q6. Of the 630 travelers stating that they were transporting meat, sausages, or other pork products from their home country or from the country where their flight stopped, 138 (21.9%) were from Eastern Europe, while 490 (78%) were from Western Europe. Of those from Western Europe, 218 (44.5%) were from mainland Italy, 130 (26.5%) from France, 60 (12.2%) from Spain, 32 (6.5%) from Poland, 32 (6.5%) from Belgium, and 18 (3.7%) from Russia.

Our analysis of responses to Q5 and Q6 is reported in a 2 × 2 matrix below (Table 2): of the travelers who were transporting meat, sausages, or other pork products (Q6 = Yes), most were unaware of ASF (526, 83.5%). Of those who were transporting pork products while being aware of ASF (104, 16.5%), 44 (42.3%) were from Spain, 26 (25%) from France, 25 (25%) from Italy, and 8 (7.7%) from the Czech Republic.

## 4. Discussion

Several factors have led to the introduction or emergence of new animal diseases and zoonoses: increased international trade in live animals and food products, the movement and mixing of wild animal species, and, in the case of vector-borne diseases, the effects of climate change. As such, demand is growing for rapid and effective surveillance and response systems that ensure the optimal use of limited human and financial resources. Many information gaps persist in this field, one of which is the risk of introducing pathogens and viruses through illegal or untraceable movements, including transport by individual passengers on ships, planes, or other means of transport. Although veterinary medicine has access to a wealth of detailed official data on the movements of farm animals, data on wild animals and the possible movements of processed products are scarce.

Of these viruses and pathogens, the global spread of ASF is of primary importance; its negative consequences are global in scope [27]. The economic and social repercussions amount to tens of billions of euros in annual losses, with potential repercussions on individual livelihoods and national food security [28]. When accounting for the various populations that can be affected by ASF, the disease can only be considered to be eradicated in the absence of outbreaks in domestic pigs or when no more cases are detected in the wild boar population [29,30]. Additionally, the risk of reintroducing the virus—even in countries that have achieved disease-free status—can be very high if not accompanied by a valid and robust surveillance system that takes all components into account [30].

Given the need to estimate the sensitivity of the Sardinian ASF surveillance system due to the recent declaration of ASFV eradication on the island, we aimed to provide critical information about the probability of reintroduction caused by illegal transportation of pork products via air travel. The information gathered will be crucial for completing a risk assessment, taking into account the risk posed by both products and live animals introduced through either legal channels or illegal or untraceable methods.

The results of the present study were obtained from a large dataset of 6525 anonymized questionnaire responses collected over a six-month period. For the first time, this study provides evidence on the potential contribution of air travelers to the risk of ASF introduction by transporting pork products for personal consumption. Notably, an extremely low level of awareness of ASF was observed among travelers, with approximately 95% of respondents declaring no knowledge of the disease. This lack of awareness was observed even among travelers from countries where ASF is currently present or has been reported in recent years. Detailed analysis by country of origin revealed notable differences in ASF awareness, with slightly higher levels among travelers from Spain, Germany, and France, and lower levels among those from Eastern European countries. Nevertheless, even in countries with long-standing ASF surveillance and control measures, overall awareness remained limited.

Furthermore, this lack of awareness was particularly pronounced among travelers who were transporting pork products, the majority of whom were unaware of ASF and its associated risks. Nearly 10% of respondents declared that they were transporting meat, sausages, or other pork products in their luggage, confirming that this practice is not marginal and may represent a non-negligible pathway for pathogen introduction. This finding is consistent with previous risk assessments that have highlighted passenger luggage as a plausible route for ASF introduction, although these were largely based on expert opinion or indirect data rather than on direct behavioral evidence from travelers [11,12,13]. However, while such associations have been extensively documented on threats to human health, comparable evidence for transboundary animal diseases has been largely scarce until now. Furthermore, it should be acknowledged that the observed prevalence of pork product transport (9.7%) is likely a conservative lower-bound estimate, as the carriage of animal-origin food products may be perceived as legally sensitive or restricted, potentially leading to underreporting despite the anonymous nature of the survey.

Our trend analysis suggests that the sample of respondents reflects the characteristics of the general traveling population in terms of time and frequency of arrivals. The majority reported traveling for holiday purposes, reinforcing the central role of tourism in shaping passenger flows to Sardinia. The pronounced seasonal pattern, with most questionnaires completed between July and September, aligns with the peak tourist season in Sardinia. From an epidemiological standpoint, this aspect is particularly relevant, as periods of intense travel are associated with an increased likelihood of introducing animal products that could be contaminated with the ASF virus.

This finding has important epidemiological implications, as leisure travelers are more likely to stay in temporary accommodation facilities—such as hotels, resorts, campsites, and holiday rentals—that are often located in coastal or rural areas rather than in densely urbanized settings. These environments are characteristic of Sardinia and frequently overlap with natural or semi-natural landscapes where wild boar populations are widely present.

The spatial convergence of tourist accommodation, natural habitats, and wild boar populations may create conditions conducive to indirect ASF virus transmission if pork products or food waste are not disposed of appropriately. Previous studies have shown that wild boar are highly opportunistic scavengers and may gain access to improperly managed waste, particularly in rural or peri-natural areas, facilitating the maintenance and spread of the ASF virus in the environment [28]. In this context, the inadequate disposal of food waste by travelers or insufficient biosecure waste management practices at accommodation facilities could act as critical interfaces between human behavior and wildlife exposure.

This scenario is especially relevant for Sardinia, where wild boar densities remain high in several coastal and inland areas, and where outdoor recreational activities and nature-based tourism are widespread. The potential access of wild boar to contaminated food waste has been repeatedly identified as a plausible mechanism for the persistence and re-emergence of ASF in endemic and post-eradication settings [31,32]. Although the present study did not directly assess waste management practices or wildlife contact, the observed combination of tourist-driven travel, transport of pork products, and low awareness of ASF highlights a behavioral pathway that warrants careful consideration within post-eradication surveillance and prevention strategies.

Taken together, these findings suggest that the risk associated with passenger-borne pork products is not limited to the point of entry itself, but may extend along the entire travel chain, from arrival to accommodation and waste disposal. Addressing this risk may therefore require integrated interventions, including traveler-targeted risk communication, awareness campaigns for tourism operators, and strengthened biosecure waste management in areas where human and wild boar populations coexist [8].

Despite its strengths, this study has several limitations. Participation was voluntary and almost entirely QR-code-based (≈99%), which may have introduced selection bias, and responses were self-reported, potentially leading to the underreporting of risky and legally sensitive behaviors. In addition, at the starting point of this study, the port authorities declined to participate, and thus the study was conducted only in airports. Therefore, we were unable to capture passenger flows arriving by sea, which may represent an additional pathway for the transport of pork products. Furthermore, considering that the questionnaires were aimed at people waiting at airports, their length was limited to five questions, even though it was clear that this would greatly reduce the amount of information that could be collected. Thus, we may have underestimated the prevalence of pork transportation and our results cannot be fully generalized to all travelers.

Many questions remain unanswered, and further investigations will therefore be necessary. Uncooked products that are improperly disposed of and eaten by feral pigs are the main area of concern with regard to ASFV. To conduct an adequate risk analysis, it remains to be determined whether the pork products being transported were raw or cooked, whether they were purchased from the original producer, and where they were purchased.

Nevertheless, our large sample size and the consistency of the patterns observed across airports and time periods support the robustness of our findings.

## 5. Conclusions

In conclusion, this study provides novel empirical evidence that traveler behavior, particularly the transportation of pork products for personal consumption, constitutes a measurable and previously underexplored risk for the (re)introduction of ASF. The identified combination of low disease awareness and non-negligible levels of pork products being transported underscores the need to integrate human behavioral data into animal disease surveillance systems. Questionnaire-based approaches at points of entry represent a promising, cost-effective tool to support risk assessment, enhance targeted communication strategies, and strengthen prevention measures in regions experiencing high volumes of travelers. Incorporating such approaches into post-eradication surveillance frameworks could significantly contribute to safeguarding ASF-free status and improving preparedness against future transboundary animal disease threats.

In addition, the absence of port authority participation should be explicitly recognized as a critical surveillance gap along sea travel routes, which warrants targeted policy attention, particularly in light of the approximately 2.5 million passengers who arrived in Sardinia by sea in 2025.

These findings clearly support the need for targeted and effective risk communication strategies at airports, particularly during peak travel periods. Improving the visibility of biosecurity messages, providing multilingual information, and engaging travelers through digital tools such as QR codes and online questionnaires could play an important role in raising awareness and promoting responsible behavior.

## Figures and Tables

**Table 1:** Summary of responses collected from questionnaires administered on a voluntary basis at Alghero, Cagliari, and Olbia airports between 1 July and 31 December 2025.

Question	Response
Q1. You are in Sardinia for	
Vacation	5530 (84.7)
Work	914 (14.0)
Study	78 (1.2)
Other	2 (0.1)
Q2. Where are you from?	
Western Europe	4094 (62.8)
Eastern Europe	2324 (35.6)
Asia	106 (1.6)
Africa	0.0 (0.0)
North America	0.0 (0.0)
South America	0.0 (0.0)
Australia	0.0 (0.0)
Q3. Did you make any flight stops to get there?	
Yes	3956 (60.6)
No	2568 (39.4)
Q4. Country from/Flight stop from:	
Italy	2802 (43.0)
Germany	1512 (23.2)
Belgium	616 (9.4)
Spain	590 (9.0)
Czech Republic	466 (7.1)
France	338 (5.2)
Poland	178 (2.7)
Russia	18 (0.3)
Switzerland	4 (0.1)
Austria	0.0 (0.0)
Denmark	0.0 (0.0)
Estonian	0.0 (0.0)
Hungary	0.0 (0.0)
Ireland	0.0 (0.0)
Latvia	0.0 (0.0)
Norway	0.0 (0.0)
Portugal	0.0 (0.0)
Slovakia	0.0 (0.0)
Svezia	0.0 (0.0)
United Kingdom	0.0 (0.0)
Q5. Do you know African swine fever?	
No	6.188 (94.9)
Yes	336 (5.1)
Q6. Have you transported meat, sausages or other pork products from your home country or from the country where your flight stopped?	
No	5894 (90.3)
Yes	630 (9.7)

**Table 2:** Matrix comparing responses to Q5 (Are you aware of African swine fever?) and Q6 (Have you transported meat, sausages or other pork products from your home country or from the country where your flight stopped?).

	Q6	
Q5	Yes	No
Yes	104 (16.5%)	232 (4%)
No	526 (83.5%)	5662 (96%)
Total	630	5894

## Data Availability

The original contributions presented in this study are included in the article. Further inquiries can be directed to the corresponding author.
